# Aortic Valve Replacement and Tricuspid Valve Repair Five Years After Orthotopic Heart Transplantation in an Adult Patient

**DOI:** 10.7759/cureus.44688

**Published:** 2023-09-04

**Authors:** Besart Cuko, Massimo Baudo, Antoine Beurton, Olivier Busuttil, Mathieu Pernot

**Affiliations:** 1 Department of Cardiology and Cardio-Vascular Surgery, Hopital Cardiologique de Haut-Leveque, Pessac, FRA; 2 Department of Cardiac Surgery, ASST Spedali Civili di Brescia, University of Brescia, Brescia, ITA; 3 Department of Cardiac Surgery, University Hospitals Leuven, Leuven, BEL; 4 Department of Cardiovascular Anesthesia and Critical Care, Hopital Cardiologique de Haut-Leveque, Bordeaux University Hospital, Pessac, FRA

**Keywords:** aortic valve replacement (avr), adult case report, surgical case reports, lung and cardiac transplantation, orthotopic heart transplantation, heart transplantation, tricuspid valve repair

## Abstract

In the current era of improved survival after orthotopic heart transplantation, post-transplant valve dysfunction is not an uncommon occurrence. The first treatment is medical management, but when it fails, surgery, possibly as retransplantation, may be needed. However, due to the scarcity of donor hearts, efforts are being made on the preservation of the cardiac allograft function by conventional operations in lieu of retransplantation. In this case report, we present a patient developing severe aortic valve insufficiency and moderate-to-severe functional tricuspid valve insufficiency five years after cardiac transplant, leading to progressive clinical deterioration and heart failure symptoms. The aortic valve was replaced with an Edwards Inspiris Resilia Nr. 23 biological prosthesis, and the tricuspid valve was repaired with a Medtronic Contour 3D Nr. 28 annuloplasty. The postoperative course was uneventful, and the patient remained well six months after her reintervention. In the literature, cases of patients undergoing valve operations following their heart transplants have already been described. However, to the best of our knowledge, this is the first case report describing a concomitant procedure of aortic valve replacement and tricuspid valve repair in an adult patient occurring five years after her cardiac transplant.

## Introduction

Improved survival after orthotopic heart transplantation (HTx) leads to an increasing number of recipients that may eventually develop various types of morbidities that limit the graft’s function [1]. Graft complications, such as allograft coronary artery disease or valvular dysfunction, can be detrimental to the quality of life of the patient, which may become refractory to medical therapy and require surgical correction. Retransplantation may not be an option for many of these patients, mostly due to a shortage of donor hearts [2]. Thus, when feasible, conventional operations to correct structural abnormalities in previously transplanted hearts are considered more frequently. Among valve dysfunction after HTx, tricuspid valve diseases are, in absolute terms, the most frequent, reaching an incidence of 84% [3,4]. On the other hand, aortic valve dysfunctions needing redo surgery are more rare conditions. The first case of aortic valve replacement was reported in 1991 by Goenen et al., 31 months after orthotopic HTx in a 28-year-old male patient [5]. The donor’s heart bicuspid aortic valve caused severe aortic insufficiency two years after transplantation [5]. From there on, redo surgeries after orthotopic HTx have been described in the literature, but conventional surgery for double or even triple valve diseases after HTx still remains a rare and challenging intervention. In this context, we report a case of successful concomitant aortic valve replacement and tricuspid valve repair performed five years after orthotopic HTx. The aortic valve was replaced with an Edwards Inspiris Resilia Nr. 23 biological prosthesis, and the tricuspid valve was repaired with a Medtronic Contour 3D Nr. 28 annuloplasty. The postoperative course was uneventful, and the patient was discharged home two weeks after the reoperation. To the best of our knowledge, this is the first case report describing a concomitant procedure of aortic valve replacement and tricuspid valve repair following an orthotopic HTx.

## Case presentation

A 58-year-old woman underwent orthotopic cardiac transplantation in 2017 for familial dilated cardiomyopathy with phospholamban mutation [6]. Her cardiological history before cardiac transplantation included a cardiac arrest in 2004 with ICD implantation thereafter. Between 2004 and 2017, multiple episodes of chronic heart failure occurred, atrial fibrillation/flutter was ablated three times, and multiple internal electric shocks were reported for syncopal ventricular tachycardia. The patient was finally transplanted in December 2017. Cardiac and coronary angiograms were carried out on the donor’s heart, which appeared to be normal. The transplantation was performed with the bicaval anastomosis technique, and no particular difficulties were encountered during the operative procedure. The postoperative course was characterized by right ventricular failure needing extracorporeal membrane oxygenation (ECMO) support for 10 days. No other relevant findings were reported till her discharge home.

Her non-cardiological history after the cardiac transplantation was characterized by amiodarone-induced hypothyroidism and chronic renal disease that progressed to end-stage, and she eventually required a renal transplantation in May 2021. Despite an episode of anti-calcineurin toxicity, the renal allograft preserved normal function before reintervention. 

At the four-year cardiological follow-up, the cardiac allograft showed a normal function. The following year, moderate dyspnea and reduced exercise tolerance developed with increasing symptomatology. She progressively worsened symptomatically with increasing severity of the aortic regurgitation. Transthoracic/transesophageal echocardiogram showed severe aortic regurgitation due to prolapse of the right coronary cusp with dilatation of the left ventricle, normal diameters of the aortic root or ascending aorta, mild-to-moderate mitral regurgitation, and moderate-to-severe functional tricuspid regurgitation (TR) with moderate dilatation of right ventricle. Cardiac angiogram and right heart catheterization did not find pathological lesions or pulmonary arterial hypertension. The functional status deteriorated from NYHA class I following the HTx to classes III and IV. No major rejection or infection episodes had occurred. After the Heart Team discussion, a redo intervention was opted for, and an aortic valve replacement and tricuspid valve repair were performed in November 2022, five years after the transplantation.

The technical details of the surgical procedure consisted of a redo median sternotomy with adhesiolysis from her previous cardiac transplant operation. After systemic heparinization, aortic and bicaval cannulation were placed to establish cardiopulmonary bypass (CPB) with moderate hypothermia (32°C). After aortic cross-clamping and oblique aortic incision, intermittent selective infusion of cold potassium cardioplegic solution into both coronary ostia was infused for myocardial protection, which was repeated every 35 minutes. The left ventricle was vented through the right superior pulmonary vein. The aortic valve was inspected and was found to have three thin, insufficient aortic cusps, especially the right one, without signs of infection or calcification. The cusps were removed, and an Edwards Inspiris Resilia Nr. 23 aortic bioprosthesis was seated. Through a right atrial incision, the tricuspid valve was inspected and was found to be of normal appearance without signs of damage. The cause of TR was the result of annular dilatation due to right ventricular dilatation, so a tricuspid valve annuloplasty with insertion of the Medtronic Contour 3D Nr. 28 ring was performed. The total bypass time was 120 minutes, and the aortic cross-clamp time was 71 minutes. The patient was easily weaned from CPB in normal sinus rhythm. Intraoperative transesophageal echocardiogram showed good biventricular function and no aortic or tricuspid residual regurgitation. Inotropic support was used for a short time after intervention. The patient was rapidly extubated and placed back on her medicinal regimen. The global postoperative course was uncomplicated, and the patient was discharged from the hospital two weeks after the operation. The six-month follow-up showed a total recuperation of NYHA class function and a well-functioning aortic bioprosthesis with mild residual TR at transthoracic echocardiogram. There have been no detectable episodes of rejection or infection after surgery.

## Discussion

The progressively longer survival of transplanted patients has shown a growing number of patients that are experiencing chronic allograft complications. This may limit the graft’s function and may be life-threatening. In most cases of valve dysfunction after orthotopic HTx, recipients are amenable to medical therapy management. In cases refractory to medical treatment for those who develop ventricular dysfunction, surgical therapy, consisting in most instances of retransplantation, should be considered [7]. However, the shortage of donors significantly hampers the organ’s availability in the current era of increasing demand [8]. Therefore, retransplantation indications must be weighed against alternative therapeutic approaches like conventional cardiac surgery following HTx. There is increasing evidence suggesting that the utilization of marginal donors yields satisfactory outcomes [9], and the objective of expanding the selection criteria is to widen the availability of donor hearts. However, not all marginal donors will have ideal outcomes, with the risk of suboptimal results.

TR is the most common valvular disease following HTx, principally due to direct damage of the valve apparatus by repeated right heart catheterizations and/or endomyocardial biopsies [10,11]. Other less frequent causes include annular dilatation and pulmonary hypertension [12]. On the other hand, aortic valve dysfunction following orthotopic HTx is a more rare condition, and apart from endocarditis, it sometimes remains with an undetermined etiology [13] or is related to a bicuspid aortic valve in the donor heart in a minority of cases [5,14]. Valve surgery after orthotopic HTx is seldom performed, and double valve surgery is even more rarely performed. Currently, guidelines on cardiac surgery after HTx are lacking, requiring individual case evaluation on the surgical indication, possibly by a specialized multidisciplinary team. As such, surgical treatment of valve pathologies after HTx remains controversial, mainly due to the limited reported evidence. The general recommendations of conventional cardiac surgery appear to be applicable in these cases while waiting for more specific indications for transplanted patients.

This case presents a concomitant double valve surgery following HTx, and to the best of our knowledge, it is the first reported case involving aortic and tricuspid valve diseases. Even though the patient might eventually need a retransplantation, she appeared to be well six months after her reoperation, with decreasing diuretic requirement and symptom improvement. Valve surgery appears to be a promising approach to extend the durability and improve the symptoms of patients with transplanted heart and valvular pathology. Open cardiac valve repair or replacement will likely gain a significant role in restoring cardiac function in post-transplant patients and allowing a lifelong allograft function.

## Conclusions

Conventional cardiac surgical procedures can be safely performed after HTx in selected cases. Surgical correction of valvular lesions is possible if the function of the previously transplanted heart is acceptable. As shown in this case report, redo conventional surgery following orthotopic cardiac transplantation may not only improve the patient’s functional status and survival but may ultimately reduce the need for retransplantation, especially in this era of donor heart shortage. Nevertheless, more precise guidelines are warranted to fill the current gap in evidence.
